# The Enigmatic Tumor Suppressor p53 Biomolecule: Its Role and Prognostic and Predictive Values in Cancer Therapy and Precision Medicine

**DOI:** 10.3390/biom16060800

**Published:** 2026-05-28

**Authors:** Zahid Hussain Siddik

**Affiliations:** Department of Experimental Therapeutics, The University of Texas MD Anderson Cancer Center, Houston, TX 77096, USA; zsiddik@mdanderson.org

**Keywords:** cancer therapy, p53 activation, post-translational phosphorylation, resistance mechanisms, MDM2, MDM4, precision medicine

## Abstract

The p53 biomolecule is critical for facile antitumor response in cancer therapy. Once fully activated, p53 will kill tumor cells by activating programmed cell death (PCD), such as apoptosis and ferroptosis, and inducing immunogenic cell death. It is not surprising, therefore, that in about 50% of cancers p53 is mutated and non-functional, which induces drug resistance. Paradoxically, many cancers harboring the wild-type *p53* genotype also become resistant via loss of drug-induced activation of p53. Efforts to convert loss-of-function or gain-of-function mutant p53 to the wild-type phenotype or to activate wild-type p53 through drug design have been disappointing. There is also a failure to recognize the existence of a sizeable number of mutant p53s that are phenotypically normal but cannot be functionally activated. Since such mutants and wild-type p53 retain intrinsic PCD pathways, focus on activating p53 could be more rewarding if the efforts implemented recognize and incorporate mechanistic factors that are vital for activating p53 function. This would realize a seminal goal of harnessing the true potential of p53 through more rational and effective therapeutic strategies and finally fulfill a critical unmet clinical need, particularly in the context of precision medicine. For such a vision, functional evaluation of normal (wild-type) or mutant p53 (or FENOMP) is vital and, thus, proposed herein as a conceptual assay to predict the phenotype of p53.

## 1. Introduction

Cancer touches everyone both physically and emotionally, whether directly as patients or indirectly as caregivers or family members. It also has a financial impact as cost of cancer care continues to spiral upwards, with an estimated annual cost in the United States alone projected to rise to about $246 billion by 2030 [1]. Most of this will be driven by the cost of actual therapeutic products, with projections estimated to reach $180 billion by the year 2028 [2]. It is likely that this estimate may well be exceeded as more and more expensive treatment options become available, especially those developed for cellular and immuno-oncology therapies, which are estimated to cost about $400,000 [3] and $185,000 [4], respectively, for each course of treatment. Despite the staggering expenses associated with cancer care, the improvement in survival rate is in general small and incremental. Moreover, there is little or no benefit realized from recent therapeutic discoveries and developments in the management of several common cancers, including pancreatic and hepatic cancers [5]. Indeed, many cancers are still dependent on traditional modalities as frontline treatment options, such as radio- and/or chemotherapy, which often fail due to therapeutic resistance from multiple cellular mechanisms that are either intrinsic or acquired [6,7]. Ovarian cancer, for instance, is usually detected late at an advanced stage, and even though it responds well to platinum-based therapy, tumor recurrences and relapse occur in up to 70% of the patients, resulting in a 5-year survival rate of only 30–40% [8]. Thus, there is a vital need to develop improved and novel options in a rational manner that are more affordable and effective. With a myriad of genetic alterations identified as potential targets for cancer treatment, and the list forever growing, the task is not easy and adds to the urgency for more effective therapeutic discoveries. Thus, it is appropriate to consider the old but well-known biomolecule p53 as a therapeutic target due to its acknowledged central role in antitumor programmed cell death (PCD) response. Theoretically, targeting p53 should be of clinical benefit against at least a half of all cancers where it is present in the wild-type state [9,10]. In practice, this is far from being true since the p53 cannot be activated functionally by the frontline therapeutic regimen. Conversely, specific mutations of p53 may not necessarily negate its function, but efforts to clinically identify such mutants are seldom pursued due to the prevailing dogma that the mutant protein is essentially non-functional. Therefore, careful consideration of our present-day knowledge of the intricate regulation of p53, including its conversion from a non-functional quiescent protein to an activated form, is critical. Such an understanding will allow therapeutic opportunities to emerge and validate the significance of p53 as a rational, feasible and viable target for more effective and low-cost cancer care. Accordingly, this review article will first summarize the salient background knowledge of p53. It will then discuss why p53 fails to function so that we can begin to fully appreciate what steps are needed to restore its therapeutic powers. Finally, this article will indicate how we may move forward to identify the functional status of wild-type or mutant p53 in tumors and, thereby, permit the design of strategies for a p53-based PCD-centric therapy in precision medicine.

## 2. Characteristics of the p53 Tumor Suppressor

The p53 protein in mammalian cells, encoded by the human *TP53* gene, was identified in 1979 and is often referred to as the “guardian of the genome” for its role in ensuring the integrity of DNA as a critical component and suppressing tumor development. The biomolecule has since been the subject of much scientific interest; a search by the author for reports with p53 or TP53 in the title resulted in 396,000 hits on Google and 47,500 on PubMed. Based on these reports, we can appreciate the vital importance and characteristics of the p53 protein and how it functions as a transcriptional factor to regulate a variety of cellular events [11]. Such events not only suppress tumors from emerging but also contribute to antitumor activity in response to therapy-induced cellular stress when cancers do emerge by evading cellular surveillance. It is, therefore, not surprising that the failure of p53 to regulate these events results in therapeutic resistance and treatment failure.

**Figure 1:**
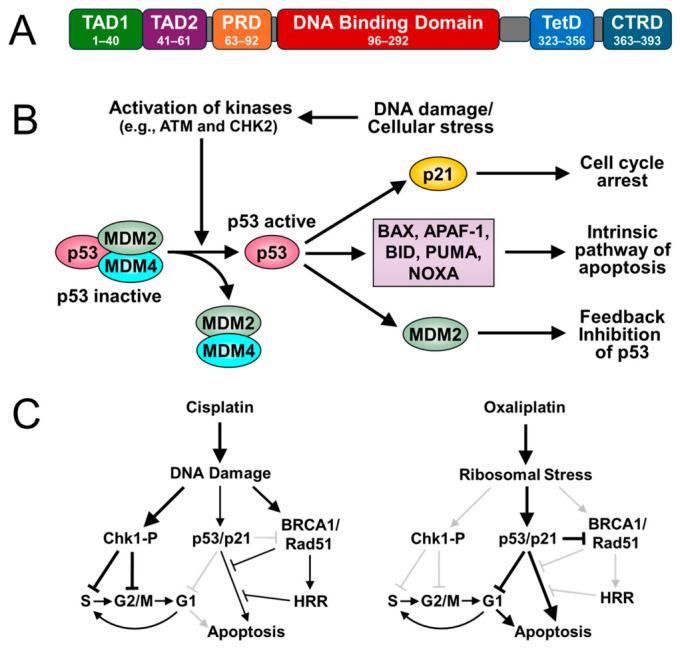
The structure of p53 and its downstream effects. (**A**) The various domains of p53 protein are shown in a linear format to simplify visualization. (**B**) p53 protein is inhibited by its binding to MDM2 and MDM4, which are released by cellular stress to functionalize p53 and transactivate target genes to regulate cellular functions, such as cell cycle arrest, apoptosis, and feedback inhibition. (**C**) Cisplatin and oxaliplatin have different mechanisms of action and, thereby, differentially upregulate the p53/p21 pathway, which in turn differentially transrepresses *BRCA1* and *Rad51*, inhibits the G1 phase of the cell cycle, and induces apoptosis, as indicated by the bold lines for robust effects, light lines for weak effects, and intermediate lines for moderate/strong effects (modified from [14]).

The human version of the protein is composed of 393 amino acids forming six distinct functional domains, namely two N-terminal transactivation domains (TAD1 and TAD2), a proline-rich domain (PRD), a DNA-binding domain (DBD), a tetramerization domain (TetD), and a C-terminal regulatory domain (CTRD) (Figure 1A). These domains regulate the function of p53 through its binding as a homotetramer to p53 response elements (p53REs) of target genes in a sequence-specific manner to transcriptionally upregulate gene expression [11]. Over 3500 potential gene targets have been identified using genome-wide analysis, but so far only about 10% of these have been verified as bona fide targets, which include the widely studied *p21*, *MDM2*, *BAX*, and *PUMA* (Figure 1B) [12]. This vast array of gene targets is consistent with the large variety of functions associated with p53 in response to cellular stress affected through diverse mechanisms, including activation of oncogenes, hypoxia, oxidative imbalance, and DNA damage [11]. However, not all genes are activated at the same time in response to a given stress stimulus, but are coordinately regulated based on such factors as the type of stress stimulation (e.g., DNA damage vs. targeted kinase inhibition), its potency, duration of exposure and mode of action, and the cell type under examination [12]. That is, each distinct stress stimulus and cell context will affect a small number of specific gene targets to achieve specific functional endpoints. This may be easier to explain using the FDA-approved cisplatin and the analog oxaliplatin as examples. Both are platinum-based anticancer drugs, with distinct mechanisms of action (DNA damage vs. ribosomal stress, respectively) that still lead to activation of p53 and transactivation of genes (e.g., the cell cycle inhibitor *p21*) as common events [13,14]. However, the observed effects of cisplatin are a slow S-phase progression, G2/M cell cycle arrest and upregulation of the homologous recombination repair (HRR) pathway, all of which attenuate the apoptotic stimulus. These effects contrast with those of oxaliplatin at an equitoxic concentration, which induces apoptotic-facilitating G1 arrest and suppression of HRR as a result of the robust induction of p21 and the lack of S- and G2/M-phase effects [14], as depicted in Figure 1C.

## 3. Regulation of Basal Levels of p53 and Its Activity

The biomolecule p53 is regulated at the protein level, and not at the transcriptional level, by suppressing its basal expression and, thereby, functions. The underlying mechanism involves the binding of p53 through its N-terminal to the N-terminal tails of the endogenous negative inhibitors MDM2 and the closely related MDM4 (also known as MDMX). The binding is strengthened through dimeric interaction between MDM2 and MDM4 via their respective C-terminal RING domain [15,16]. In this manner, the p53 within the heterotrimer complex is held tightly in an inactive state (Figure 1B). Moreover, these inhibitors through the E3 ubiquitin ligase function of MDM2 induce poly-ubiquitination of p53 at the lysine residues, which promotes constitutive p53 degradation through the proteasomal pathway to maintain the tumor suppressor at the low level. Evidence from transgenic mice demonstrates that the interaction between MDM2 and MDM4 is vital for suppressing p53 since the C462A RING domain mutant of either inhibitor prevents this interaction and also suppresses E3 ligase function, which then stabilizes and activates p53, resulting in embryonic lethality that is rescuable by deleting p53 [17]. Notably, the RING domain mutants can still bind to p53, which prompts the conclusion that the trimeric complex bound through only the N-termini is unable to suppress p53 function [18]. Homodimers of MDM2 can also bind to p53 and ubiquitinate it for degradation, but MDM4 homodimers cannot degrade p53 as they lack E3 ligase activity [16]. This is an important understanding as it establishes a mechanism for MDM2 transactivated by functionalized p53 to turn off the tumor suppressor through feedback inhibition.

## 4. Stabilization and Activation of p53

Both MDM2 and MDM4 are important in suppressing p53; this has been elegantly demonstrated by *MDM2* or *MDM4* knockout in mice and validated by rescuing the resulting embryonic lethality following simultaneous knockout of p53 [19]. In specific terms, based on data from genetically engineered mouse models, MDM2 has been proposed to regulate the stability of p53, with MDM4 regulating the transactivation of p53 [20]. Other evidence also indicates that the phenotypic response in MDM2 knockout cells is distinct from that in MDM4 knockouts [19]. Interestingly, recent data supports the concept that the main inhibitor of p53 is essentially MDM2 and that MDM4 contributes by modulating MDM2 inhibitory activity [21]. This does not negate the fact that from a therapeutic perspective targeting either MDM2 or MDM4, such as with siRNA [22], will stabilize and activate p53. It is important to note that wherever possible for clarity in this review, stabilization and activation are used as two separate terms in reference to p53: since the turnover of p53 protein is rapid, stabilization is used to indicate increases in protein levels, whereas activation is used when transactivation of downstream target genes is affected. This is appropriate as stabilization may induce low-level activity but this does not occur in a consistent manner, as has been observed in resistant tumor cells where cisplatin-induced increases in p53 levels do not result in significant target gene upregulation [22].

**Figure 2:**
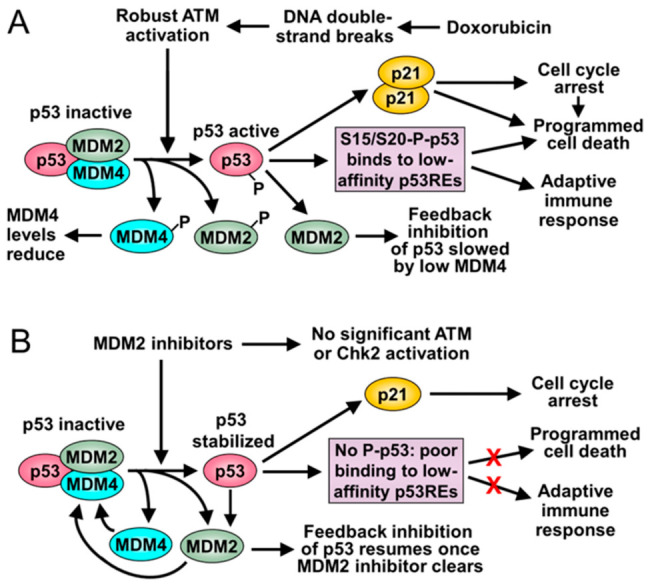
The level of stabilization and/or activation of p53 and the impact of phosphorylation are drug-dependent. (**A**) Doxorubicin induces DNA double strand breaks, resulting in activation of DNA damage response pathways, which includes ATM-dependent phosphorylation of p53, MDM2, and MDM4 and reduction in the levels of the latter protein due to degradation. The entire process results in robust p53 upregulation that transactivates genes inducing cell cycle arrest, feedback inhibition, programmed cell death (e.g., apoptosis), and adaptive immune response, which may possibly trigger immunogenic cell death (ICD). The robust increases in p21 also contribute to cell death by facilitating p53-dependent transrepression of anti-apoptotic genes. (**B**) MDM2 inhibitors, such as nutlin-3a, stabilize and partially activate p53, which modestly induces p21 for cell cycle arrest and MDM2 to increase the pool for feedback inhibition; there is general absence of phosphorylation of p53 and endogenous inhibitors, p53-dependent apoptosis, and adaptive immune response at the physiological drug concentrations.

Therapy-mediated stabilization and activation of p53 is an intricate and orchestrated process and central for the effective management of cancer patients. The antitumor modalities and the cellular stress generated in tumors as a result, first activate several signal transduction pathways that converge on the heterotrimeric complex to then post-translationally modify p53, MDM2, and MDM4. This uncouples, stabilizes, and activates p53, which then translocates to the nucleus to undertake transactivation function. To accomplish this goal, the p53 molecule is subject to different types of modifications, including phosphorylation, methylation, ubiquitination, acetylation, sulfation, NEDDylation, SUMOylation, and hydroxylation [23]. Of these, phosphorylation is the most critical in disassembling the trimeric complex [24], as shown in Figure 2A, and the most studied regarding cancer cause and treatment. The salient points on phosphorylation of the three central players are covered in the following sub-sections.

### 4.1. MDM4

Phosphorylation of MDM4 induces degradation and reduces its levels and, thereby, promotes the disassembly of the trimeric complex. This has been amply demonstrated in cells exposed to DNA-damaging agents, such as UV, doxorubicin, and ionizing radiation, which activate several kinases, including ATM, Chk2, Chk1, and AMPK. These kinases mediate phosphorylation of human MDM4 at a number of serine sites that include Ser342, Ser367, and Ser403, some of which facilitate the ubiquitination and proteasomal degradation of MDM4 in an MDM2-dependent manner [25,26,27,28,29,30]. This is consistent with evidence from transgenic mice that phospho-resistant Ser-to-Ala mutations at the Ser-sites of murine MDM4 corresponding to the above three sites still allows binding to MDM2 but protects MDM4 from degradation and, thereby, increases radioresistance in mice [15,31]. Since sufficient reduction in MDM4 levels is considered a key for p53 to achieve maximal activation [15,20,32], cells can also utilize an additional mechanism to affect or supplement this reduction, namely transcriptional inhibition, as has been demonstrated with cisplatin and doxorubicin [33]. Based on observations that the CDK4 inhibitor and antitumor agent palbociclib suppresses MDM4 transcripts [34], it is likely that transcriptional inhibition of MDM4 by DNA-damaging agents is a secondary event downstream of p53 and mediated through p21. Thus, p21-dependent CDK4 inhibition in G1 phase of the cell cycle will sustain or even elevate p53 activity to exceed the threshold for its programmed cell death function.

### 4.2. MDM2

Stress-induced modification sites of MDM2 are numerous and include Ser386, Ser395, Ser407, Thr419, Ser425, and Ser429, phosphorylation of which has been reported to suppress the various mechanisms that inhibit p53 function [24,32,35]. Specifically, ATR-mediated phosphorylation at Ser407 following exposure to UV stabilizes p53 by inhibiting its export from the nucleus to the cytoplasm [35], whereas ATM-mediated phosphorylation at Ser386 and Ser429 with ionizing radiation inhibits the ability of MDM2 to oligomerize via the RING domain and, thereby, prevent ubiquitination and degradation of p53 [32]. In an experimental demonstration, MDM2 phosphorylation at all six sites identified above provides the greatest p53 stabilization, which further supports the notion that the MDM2 RING domain is a major modulator of p53 function [32].

### 4.3. p53

Phosphorylation of p53 can occur at about 20 different sites, but not all at the same time. The sites that get phosphorylated are dependent on the mechanism of action of the stress-inducing stimulus [36]. In several cases, each site can be targeted by multiple kinases, as exemplified by the Ser15 site that can be modified by at least eight different kinases [36]. Although these many kinases may seem highly redundant, it is again likely that not all kinases are activated simultaneously; each stress-inducing agent may stimulate distinct signal transduction pathways that activate kinases in a selective manner to phosphorylate p53. Moreover, it is worth noting that a single kinase can target multiple sites in p53, as is the case with Chk2, which can target five different sites and in parallel also phosphorylates MDM4, but it is interesting that MDM2 with a close homology does not appear to be a target of this kinase [15,24,25,26,27,28,29,36,37]. The multiple kinases and phospho-sites, therefore, confound a good understanding of which combinations and permutations of p53 phosphorylation are critical for its robust activation and cell-killing functions for effective cancer therapy. However, attention has been focused on the N-terminal transactivation domain (TAD 1) section of p53 for three reasons: (1) the N-terminal tails of MDM2 and MDM4 bind to this section and mask the evolutionary conserved Ser15 and Ser20 phospho-sites to block transcriptional function of p53 [38]; (2) phosphorylation of Ser15 and Ser20 weakens the binding between p53 and MDM2 and MDM4 and contributes to the disassembly of the complex [39]; and (3) phospho-resistant S15A- and/or S20A-p53 mutants in mice induce spontaneous tumors [38]. Interestingly, phosphorylation of Ser15 is considered a priming event for Ser20 phosphorylation [39]. Moreover, Chk2 defects impact Ser20 phosphorylation and transactivation of p21, but not Ser15 phosphorylation and p53 stabilization [22]. Therefore, it is likely that the Ser20 site has a more critical role in p53-mediated transactivation and Ser15 in p53 stabilization. While there is general consensus over this role of the Ser20 site [40,41,42], there is some healthy disagreement [38].

Following the orchestrated phosphorylation of p53, MDM2, and MDM4, it is rational to acknowledge that the modified p53 will be released from its inhibited trimeric complex to execute its functions, with transactivation of nascent MDM2 providing an important means to limit p53 activity by feedback inhibition. It is important to also accept that this feedback inhibition must apply to all active forms of p53, including the phospho-forms. In this regard, while it may be demonstrated that phosphorylation of the Ser20 site in activated p53 blocks its binding to MDM2 [43], protein–protein interactions are still governed by concentrations and the increasing levels of transactivated MDM2 will eventually shift the interaction equilibrium toward increased binding to suppress the active p53.

## 5. Phosphorylation, p53 Dynamics, and Cell Fate

Whether phosphorylation of p53 is needed for p53 stabilization and activation, and at which sites, has been an ongoing debate. In a similar manner, how exactly p53 decides between cell cycle arrest and programmed cell death (such as apoptosis) remains an open question. Phosphorylation induced by genotoxic agents is indeed important in the disassembly of the trimeric p53-MDM2-MDM4 complex to stabilize and activate p53 functions. However, these effects can be reproduced with non-genotoxic MDM2 inhibitors, such as nutlin-3a, that do not rely on phosphorylation to accomplish the same goal of uncoupling p53 from the inhibited trimeric complex [44,45]. Interestingly, immunoprecipitation has demonstrated that nutlin-3a not only inhibits MDM2-p53 binding, but also disrupts MDM4-p53 binding, leading to the conclusion that MDM2 inhibition destabilizes the entire complex [44]. In this manner, the MDM2 inhibitors stabilize p53 and facilitate transactivation of p21 to induce G1 cell cycle arrest (Figure 2B); however, such inhibitors lack the capacity to induce apoptosis at physiological concentrations [44,46,47,48,49]. G1 arrest alone by MDM2 inhibitors has been observed in both animal tumor models and patients [50,51], resulting in poor/modest antitumor responses that culminate in acquired resistance to the inhibitors in patients through the selection of mutant p53 clones [48,52,53]. Current indications are that MDM4 or dual MDM2/MDM4 inhibitors also induce cell cycle arrest and no apoptosis [54,55,56]. Thus, phosphorylation of p53 may not be needed to transactivate p21 and induce cell cycle arrest in specific cases but appears to be vital in executing the cell death program and inducing adaptive immune response, as is evident with doxorubicin (Figure 2A) and oxaliplatin (see below).

Other attempts to explain how p53 differentiates between cell cycle arrest and apoptosis have been offered through modulation of its dynamics. Simply put, low levels of p53 activation leads to cell cycle arrest whereas high levels exceed a specific threshold to induce apoptosis [57]. This generalization is consistent with the concept based on reaction dynamics that low levels of p53 will interact only with high-affinity p53REs, such as in the p21 gene for cell cycle arrest, whereas higher levels of p53 will facilitate interaction with low-affinity p53REs, such as in the *BAX* gene for apoptosis [58]. Further insight into the modulation of p53 functions has been provided by pulsatile stabilization of p53. In this manner, DNA-damaging agents stabilize p53 in short repeat pulses, which are orchestrated by the MDM2 feedback loop and lead to cell cycle arrest and DNA repair. If the dose of the agent is high and/or repair is futile, further stabilization of p53 results from, for instance, phosphorylation, which then switches the pulsatile oscillations of p53 levels to a sustained high level, resulting in the higher functions of apoptosis and adaptive immune response as the final outcome [59,60].

It is important to recognize that the pathway toward cell cycle arrest and cell death are not independent but intimately linked. The p21 transactivated by p53 is not only the most critical player in G1 arrest [61,62,63], but also a likely trigger for cell death if there is a system failure to rectify the cellular stress, such as that resulting from excessive DNA damage. It is, therefore, rational to also consider p21 as a tumor suppressor, which is consistent with evidence that p21 knockout in mice induces spontaneous tumors [64]. From the perspective of therapy, tumor models are more sensitive to antitumor agents if they retain an intact G1 arrest machinery [65,66,67,68] or if G1 arrest in mutant p53 tumor cells is restored by ectopic p21 expression [69,70]. Furthermore, small molecule inhibitors of G1 phase CDKs not only arrest cells but also induce apoptosis [71,72]. A seminal class is the CDK4 inhibitors [73], with the representative palbociclib demonstrating that G1 arrest is associated with downstream suppression of PRMT5 activity. As a result, interference of pre-mRNA splicing of MDM4 is induced, which leads to reduced MDM4 protein expression and subsequent p53 activation [34]. This provides a potential mechanism for the transition of cell fate from cell cycle arrest to cell death, but only if p21 upregulation is robust, as is known with doxorubicin (Figure 2A). Thus, cellular stress initially induces p53, which transactivates p21 and results in G1 arrest by inhibiting CDK4, leading to downstream signaling that suppress MDM4 expression. This suppression will further stabilize and/or activate p53 to finally exceed the threshold for inducing programmed cell death.

## 6. The Many Functions of p53

In much the same way as how cell cycle arrest or apoptosis is determined as the final fate, the other cellular functions of p53 are likely affected similarly and dictated by p53 dynamics and tightly modulated by the type, degree, and duration of cellular stress. With just over 350 gene targets of p53 identified and over 3000 potential targets yet to be investigated [12], the range of specific functions associated with this biomolecule alone is substantially broad. Even though there is still much to be discovered, the array of p53 functions uncovered over the past four decades is astounding. In addition to cell cycle arrest, DNA repair (for genomic integrity and stability), and programmed cell death that have been mentioned above, other functions include senescence, reproduction, aging, stem cell dynamics, metastasis, cell metabolism, and immunity [11,23], some of which are associated with tumor suppression or successful cancer therapy. Apoptosis is perhaps the most important in therapy and well-studied, but it is not the only form of the cell death program that is regulated by p53; there are several others and include autophagy, ferroptosis, pyroptosis, cuproptosis, and necroptosis [11,74]. Immunogenic cell death (ICD) is another form that is seminal in that it is affected by an adaptive immune response, which has the potential to provide long-term antitumor benefits by preventing tumor recurrence [74,75,76]. These additional cell death programs also have a highly critical role in antitumor response to therapy, but again which program(s) get(s) activated is dependent on the stress-inducing agent and the intensity of p53 response. For instance, only apoptosis is induced by the DNA-damaging agent cisplatin but in contrast both apoptosis and ICD are activated by the ribosomal stressor oxaliplatin [77,78,79,80]. The higher-level effect of oxaliplatin may relate to its distinct mode of action that translates to a greater intensity of p53 activation, which allows it to bind low-affinity p53REs [14], similar to that seen with doxorubicin (see Figure 2A).

## 7. Mutations Do Not Necessarily Inactivate p53 Functions

We can begin to appreciate that the p53 protein in its wild-type conformation is critical for ensuring tumor cells retain high drug sensitivities that lead to a robust antitumor drug response [65]. This is accomplished by p53 binding to p53REs and transcriptionally activating downstream target genes directly involved in programmed cell death, such as apoptosis [81], and include *BAX*, *NOXA*, *APAF-1* (apoptotic protease-activating factor 1), *PUMA*, and *BID* [82], as shown in Figure 1B. p53 can also lower the threshold for cell death by transrepressing other specific genes, such as *Rad51* and *BRCA1*, to inhibit homologous recombination repair [14,44], as represented in Figure 1C, or *BCL-2*, to diminish the anti-apoptotic influence [83]. These transrepressive effects may be an indirect effect of p53 mediated through transactivation of other genes, such as p21 [84]. Since p53 is a critical mediator of cell death, there is continued pressure for clonal outgrowths of tumor cells that lack the programmed cell death machinery. A simple and highly effective solution for cells to survive stress and become tolerant is by p53 becoming mutated in the DNA-binding domain, which alters the conformation of the tetrameric p53 that inhibits transactivation by preventing its binding to p53REs.

Mutation of p53 is the most frequent genetic defect in human tumors, impacting 50% of all cancers [9,10]. Mutation frequency, however, depends on the tissue type and can range from 2 to 3% in thyroid carcinoma to ~90% in esophageal carcinoma [85]. About 60–80% of the mutations are missense and those most frequently occurring in the DNA-binding domain (~30%) are located at the “hot-spot” sites, namely positions 175, 220, 245, 248, 249, 273, and 282 [86]. Mutations at these seven sites are highly impactful since the mutants formed may not only lose normal p53 functions but also acquire abnormal “gain-of-functions.” For instance, unlike wild-type p53, mutants R248W- and R273H-p53 acquire the ability to interact with the Mre11 protein and inhibit the Mre11/Rad50/NBS1 complex, which normally activates ATM following DNA double strand breaks [87]. “Gain-of-function” mutants can also upregulate pro-survival or tumor-growth-promoting genes, such as *BAG-1*, *EGFR*, *HSP70*, *NF-kB*, *YAP1*, and *NFKB2* and enhance resistance of tumor cells to therapeutic agents [86,88,89,90,91].

**Figure 3:**
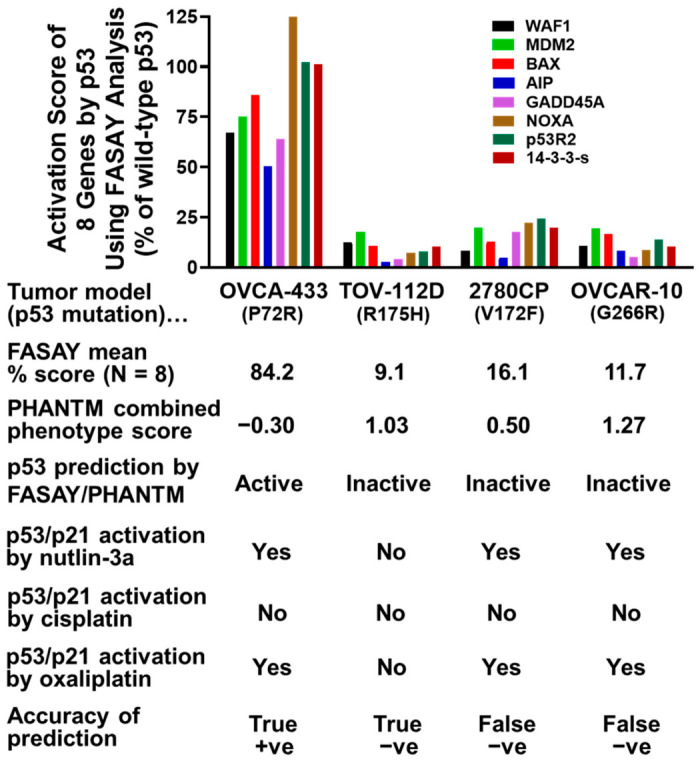
Limitations of databases for predicting the function of mutant p53. Four tumor models, three with mutant p53 and one with a P72R polymorphic mutation, were assessed for prediction of p53 function using two publicly available databases and compared to actual data reported of p53 response to nutlin-3a, cisplatin, and oxaliplatin. The TOV-112D has a hot-spot mutant p53, which is not activated by the drugs, whereas the other three tumor models lack Chk2, which prevents phosphorylation-dependent activation of p53 by the DNA-damaging agent cisplatin but is activated by nutlin-3a and by alternative kinases induced by the ribosomal stress-inducing agent oxaliplatin. The transcriptional activation of 8 genes by mutant p53 in the bar graph were obtained using the FASAY database [95], whereas the FASAY mean score were calculated from individual scores for the 8 genes and the PHANTM combined phenotype scores were obtained from the NCI website (https://tp53.cancer.gov, 12 March 2026). The data for nutlin-3a, cisplatin, and oxaliplatin and p53 mutations in the tumor models have been reported [44,100,101,102,103], except that the response to nutlin-3a in OVCAR-10 is unpublished preliminary data. +ve, positive; −ve, negative.

Since p53 mutations are frequently associated with poor therapeutic outcomes, it has led to the notion that any mutation in p53 will have a negative clinical impact. This, however, is a misconception that requires careful scrutiny. Several studies have categorized p53 mutants into groups based on relative transcriptional activity. Using a yeast-based gap repair assay (or FASAY [92]), one study examined over 2300 possible p53 mutants and found that 55% were fully transactive against eight different p53-responsive gene promoter constructs [93], as exemplified by the bar graph showing transcriptional activation of the eight genes by the P72R-p53 mutant in Figure 3. Similarly, about 30% of missense mutants in breast cancers were identified as functional [94]. Another study used the FASAY database [95] to examine the functional status of mutant p53 in colorectal cancers, and reported that about a third of mutants in cancers of the proximal colon (36%), distal colon (37%), and rectum (28%) were functionally transactive [96]. Unfortunately, the prognostic value of the mutants could not be validated as both active- and inactive-p53 tumors demonstrated similar therapeutic outcomes with the standard-of-care treatment in the clinic [97]. In order to improve predictions, a PHANTM database (https://tp53.cancer.gov, 12 March 2026) was launched based on an extensive set of published data [98]. PHANTM predicts that p53 is likely to be functional if the combined phenotype readout score of the mutation is −1 to ~0.5 and non-functional if the score is ~0.5 to 2, with low confidence in the range of ~0 to ~0.6. Using this criteria, one clinical report on myelodysplastic syndrome demonstrated that the group of mutant p53 cases with a score <1 (thus, likely includes functional and non-functional p53) was associated with a significantly greater overall survival than the group with a score >1 (median 19.5 vs. 10.6 months; *p* < 0.05) [99]. However, the utilization of this database in reports has been low, which is also the case with the FASAY database. It is possible that the predictive value of these and other databases is limited and that refinements are necessary. Figure 3 highlights a likely limitation: it indicates that p53s in cisplatin-resistant ovarian tumor models TOV-112D (with a hot-spot non-functional R175H p53 mutant [100]) and OVCA-433 (with a functional polymorphic mutation P72R) are correctly predicted by the databases; however, the predictions for p53 mutations in 2780CP (V172F) and OVCAR-10 (G266R) are incorrect (false negatives) as deduced from comparing with the reported p21 upregulation by the p53-dependent oxaliplatin and nutlin-3a [44,101]. Failure of p53 to become activated by cisplatin in the three models responsive to oxaliplatin and nutlin-3a is, therefore, not due to the mutations but due to the loss of Chk2 (discussed below). Notably, the false-negative predictions for mutant p53, albeit in only two tumor models in this demonstration, are a sufficient concern that requires recalibration of the databases for greater robustness or consideration of an alternative approach for better predicting mutant p53 function.

## 8. Status of p53, Therapeutic Response, and Resistance

Tumor cells expressing wild-type p53 are expected to be sensitive to antitumor agents [65], and this is evident in testicular cancers, which predominantly harbor wild-type p53 and are, therefore, curable with platinum-based therapy [104]. Such observations are consistent with robust activation of p53 as being sufficient to induce cell death, even when deregulated survival pathways are active in the same tumor cell [105]. Unfortunately, inactivation of p53 functions renders tumor cells resistant to therapy, both clinically and in model systems [106,107,108,109,110]. Interestingly, loss of p53 function induces cellular resistance to a wide spectrum of unrelated FDA-approved antitumor agents [111]. This emphasizes the importance of being aggressive early in cancer therapy to take advantage of the presence of functional p53 before it becomes dysfunctional.

That bona fide loss-of-function mutation in p53 will confer drug resistance and treatment failure is best exemplified with the choroid plexus carcinoma, which is clinically curable with therapy when p53 is present as the wild-type, but patients die within 6 months if p53 is mutated [112]. Similarly, about a half of all patients with advanced breast cancers respond to paclitaxel or the 5-fluorouracil/epirubicin/cyclophosphamide combination when wild-type p53 is expressed in tumors, whereas responses are negligible in the presence of mutant p53 [113,114]. In an analogous manner, the 5-year survival rate in chronic lymphocytic leukemia (CLL) patients is 3-fold greater with wild-type p53 than mutant p53 (59% vs. 20%) [115]. However, there are examples that defy expectations. One such example is the experimental OVCAR-3 ovarian tumor model: It harbors a “hot-spot” R248Q missense p53 mutation, which has a low FASAY activity score of 0.21 and a PHANTM combined phenotype score of 0.81 and, as expected, induces resistance to cisplatin in vitro [116]. Paradoxically, the tumor model as a xenograft in mice is sensitive to cisplatin administered at 8 mg/kg as the tolerated dose [117]. This anomaly may be explained pharmacologically since a pharmacokinetic report indicates that even a lower dose of cisplatin (4 mg/kg) results in plasma drug concentrations in mice of >3 µM during the first four hours [118] that exceeds the in vitro IC_50_ concentration (1.2 µM) required to kill 50% of OVCAR-3 cells [116]. Such an unexpected therapeutic response of cancers expressing mutant p53 has also been noted at the clinical level. For instance, high-grade serous ovarian cancer (HGSOC) sub-type, representing 70% of all ovarian cancers, has a 96% frequency of p53 mutations [119], but surprisingly 60–80% of the cases respond to platinum-based therapy [120], resulting in a ~40% 5-year survival rate [121,122]. These results may also be explained in part by considering that specific mutant p53 proteins may retain wild-type p53 function, which has been noted in ovarian and colorectal cancers [96,101]. Nevertheless, such observations with unexpected outcomes make it difficult to use mutations in p53 for prognostic and predictive value in the clinic, as has been highlighted in a consensus report [123].

Although the foregoing discussion suggests that wild-type p53 cancers are responsive to therapy on a consistent basis, this is usually not the case as can be highlighted by referring again to ovarian cancer. As surprising as it is to observe a good response to therapy in HGSOC despite expressing predominantly mutant p53, it is equally surprising that an identical platinum-based regimen is less effective against six rare cancer sub-types representing 30% of the total ovarian cancers and harboring predominantly wild-type p53 (~90%). Specifically, the response rates in mucinous and clear cell as two representative rare sub-types are 26 and 15%, respectively, vs. 60–80% in HGSOC [120,121]. Similar low response rates have also been observed in other cancers harboring wild-type p53, such as mesothelioma with a ~90% incidence of wild-type p53 but only a 5–33% response rate [101]. The unexpected higher response rate in ovarian cancer when mutant p53 is present compared to wild-type p53 has also been noted in other cancers. For instance, this is evident in non-small cell lung cancer (NSCLC) treated with paclitaxel (75 vs. 47%), metastatic bladder cancer treated with platinum-based combinations (50 vs. 27%), and advanced breast cancer treated with paclitaxel (83 vs. 38%) even though these therapeutic agents are dependent on wild-type p53 [111]. Thus, cancers with a wild-type p53 genotype are not just resistant to therapy but appear to be more so than mutant p53 cancers and this limits the prognostic and predictive values of both wild-type and mutant p53 genotypes in cancer therapy.

Several mechanisms can hinder the activation of wild-type p53, but the two most prominent are enhanced degradation of p53 and defective post-translational phosphorylation. The increased degradation through the ubiquitin-dependent proteasomal system can occur through the upregulation of specific proteins, such as the E6 product of human papillomavirus HPV-16 commonly found in cervical cancer [124,125]. Deregulation of MDM2 and MDM4 in cancers can lead to overexpression of these proteins that also contribute to p53 degradation [101]. A low-level 2-fold increase in MDM2 expression appears to be sufficient to prevent p53 activation [15] and reported to reduce the 10-year survival rate in breast cancer patients (58–61 vs. 73%) [126]. Overexpression or amplification of MDM4 in 10–20% of the cases can also promote p53 degradation by stabilizing MDM2 and contributing to therapeutic resistance in cancers [36,127]. Deregulation of MDM2, MDM4, and p53 through defective phosphorylation is the second mechanism that also hinders p53 activation. A notable observation under normal conditions is the drug-induced phosphorylation-dependent degradation of MDM4 and/or inhibition of MDM2 oligomerization to stabilize p53, which as discussed earlier are desirable traits for the success of cancer therapy. With loss of ATM function in CLL [128], for instance, MDM2 phosphorylation and, therefore, oligomerization via the RING domain is inhibited [32] together with loss of MDM4 degradation [25,26,27,28,29] and of the therapy-sensitizing phosphorylation of Ser15 in p53 [36]. Similarly, defective Chk2 expression in cisplatin-resistant ovarian cancer cells inhibits the phosphorylation of p53 at the key activating Ser20site [101] and of MDM4 to prevent its degradation when these cells are challenged with cisplatin [129]. A number of other mechanisms that attenuate the activation and function of wild-type p53 and, thereby, induce therapeutic resistance in a variety of cancers at both the clinical and preclinical levels were summarized in an earlier review article [91].

## 9. Targeting Wild-Type p53 Function and the Economics of Cancer Care

Cancer is a conglomeration of over 100 different diseases, each from its own tissue of origin with a unique genetic signature and distinct molecular targets that require specific treatments. Even cancers originating from the same tissue may be further sub-divided based on cell type that further undermines the possibility of a simple unified approach to cancer care. Thus, novel cancer therapeutics are developed to address the needs of patients with common cancers where the return on investment in research and development is meaningful, and this unfortunately limits efforts in developing therapeutics for rare cancers. It is, therefore, not unexpected that precision cancer medicine may remain an unrealized goal except for cancers or targets that occur frequently. In this regard, drug discovery efforts have been directed toward targeting mutant non-functioning p53 to convert it to an active form, such as with CP-31398 and PRIMA-1 [130]. However, the task is made difficult since each specific mutation appears to require a specific interacting small chemical molecule to restore wild-type function [131], and targeting the many possible p53 mutants becomes an impractical and economically burdensome proposition.

The vast body of literature provides evidence that wild-type p53 and its robust activation by antitumor agents should induce a durable therapeutic response in the clinic. This is possible since activated p53 acts as a catalyst to lower the threshold for cell death. Thus, wild-type p53 is an obvious target for therapy, but, as discussed above, treatment based on the p53 genotype does not appear to currently offer a practical solution for treating patients. Such impracticality also extends to cancers with mutant but phenotypically normal p53, which is likely compounded by an awareness that the presently available databases for predicting the functionality of mutant p53 are also not sufficiently robust to be useful as clinical tools in this endeavor. This may be due in part to the inability of databases to consider molecular factors that hinder p53 activation, such as the status of MDM2 or MDM4 expression and of the post-translational phosphorylation kinases ATM and Chk2 [98]. Indeed, many classical FDA-approved antitumor agents in clinical use today are dependent on ATM and/or Chk2 to activate p53 and induce cell death [111]. Therefore, it is reasonable to propose that the p53 mutants predicted by the database as being functional may lead to a false-positive outcome in ovarian, CLL, and similar cancers that lack these kinases. As may be expected, such a result would also be replicated in wild-type p53 tumor cells in absence of these kinases. This is supported by the finding that in at least 37% of 335 cases of epithelial malignancies the defects in p53 and either Chk2 or ATM were mutually exclusive, and that defects in both the p53 and the kinase were rare (<1%) [132]. Thus, defects in ATM and Chk2 frequently undermine the goal of inducing a robust response in tumor cells harboring either functionally mutant or wild-type p53.

In the context of functional p53 being present in tumor cells without the activating ATM or Chk2 kinase, the clinical use of non-genotoxic MDM2 and MDM4 inhibitors can be considered an option. Indeed, they have shown effectiveness in stabilizing and activating p53 in cisplatin-resistant tumor models that are Chk2-deficient and also have high levels of MDM2 and MDM4 bound to p53 [44]. However, such small molecule inhibitors only partially activate p53 since they merely induce growth arrest and not cell death at the doses used (see Figure 2B). Such a scenario in the clinic has led to outgrowth of tumor cells expressing mutant p53 and, thereby, drug resistance [48,52,53]. This impediment likely arises from a lack of consideration of molecular factors that are essential to activate p53 for its apoptotic function, as discussed above. Thus, it is not surprising that rational strategies designed for p53-targeted therapies are facing shortcomings during the clinical translation phase. It is, therefore, vital that an alternative strategy is developed that overcomes such limitations and robustly activates functional mutant and wild-type p53 so that the vision for the prognostic and predictive apoptotic value of p53 may be realized. Such a strategy that embraces an agnostic tumor type approach and potentially leads to a unified treatment of >50% of the cancer patient population would revolutionize cancer treatment as it would also be highly cost-effective due to the economy of scale. But is this realistic and realizable?

**Figure 4:**
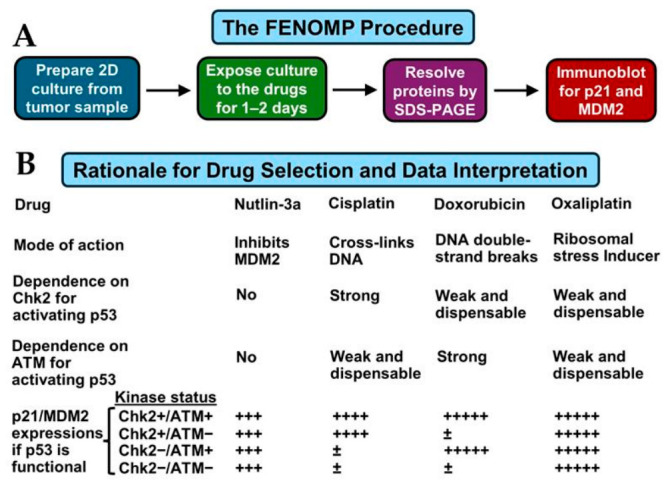
Proposed concept for functional evaluation of normal (wild-type) or mutant p53 (FENOMP). (**A**) In the simplified procedure, 2D cultures are prepared from tumor samples and then exposed to the suggested concentrations of nutlin-3a (5 µM [44]), cisplatin (5 µM [44,101]), doxorubicin (0.5 µM [145]), and oxaliplatin (5 µM [101]) for 1 or 2 days, and then probed for p21 and MDM2 through Western blot. (**B**) The mechanism of action of the four proposed drugs and response based on status of Chk2 and ATM that activate p53 are shown; a positive functioning p53 is identified through the indicated expressions of p21 and MDM2 induced by each drug depending on the presence (+) or absence (−) of Chk2 and ATM. Anticipated relative intensities of p21 and MDM2 expressions, which will vary between tumor samples due to factors such as prior treatment, are indicated as follows: ±, none/negligible; +++, moderate; ++++, strong; +++++, robust. The indicated robust expressions of p21 and MDM2 with doxorubicin and oxaliplatin are anticipated since these drugs are also inducers of immunogenic cell death [80], which requires a higher level of p53 activation.

One possible practical approach is to make use of tumor biopsies or samples that are routinely taken from patients. Such an undertaking is already of great interest for ex vivo evaluation of tumor drug sensitivity using a variety of tumor model systems for the purposes of precision medicine [133,134,135,136,137,138,139]. Thus, the methodology currently exists and adapting tumor model systems to assess the functionality of p53 ex vivo is feasible; a simple conceptual approach would be to expose a 2D tumor model for 1–2 days to rationally selected therapeutic drugs that would interrogate whether the p53 is functional and if it is Chk2- or ATM-dependent. Based on the author’s preclinical research experience, an assay procedure (FENOMP) is proposed in Figure 4A, and a set of four drugs and their concentrations, with possible expressions of p21 and MDM2 as a signature of p53 function, is suggested in Figure 4B. The suggested drugs include doxorubicin and oxaliplatin for their ability to induce immunogenic cell death through robust p53 activation [80]. If the p53 is determined to be functional and its kinase dependency and level of activation are evident from the signature, then the next step for precision medicine in an oncology setting would be to design a drug regimen that simulates maximal p53 activation ex vivo before treating the patient. Based on reports, the turnaround time could be as low as 5–10 days depending on whether the tumor is liquid or solid [135,137]. Screening of larger numbers of samples or expanding the number of p53 targets is feasible and could be automated through, for instance, the high throughput reverse phase protein array [140] or the JESS Simple Western system [141,142]. Indeed, any analytical platform to assess the expression of p53-dependent targets to replace immunoblots, such as LC-MS/MS, qPCR, or NanoString [142,143], may be incorporated into the procedural design. Refinements to the procedure, such as 3D models, tumor-on-chip, determination of combination index, microarrays, and PDX models [134,135,136,138] for analysis of tumor drug sensitivity, are appropriate but may extend the estimated turnaround time and perhaps add an unnecessary layer of complexity to the task of simply determining the functional status of p53. Whichever procedure is adopted, it is worth noting that there will be variability between tumor samples in their gene expression signatures due to the presence of a number of unknown intrinsic and/or acquired mechanisms of resistance, such as defective drug transport [144]. It is also appropriate to indicate that presence of subclones harboring non-functional p53 may reduce the predictive value of the strategy. Nevertheless, p21 and MDM2 upregulations with only one of the four proposed drugs, or even another test agent, may likely be sufficient to indicate that p53 is functional and may facilitate antitumor response, even though it may be partial due to the possible impact of non-functional p53 in subclones. The important point to convey is that the functionality as opposed to the genotype of p53 is the more important indicator of treatment response and it is feasible to determine the functional status of p53 with a high level of confidence to more rationally and effectively guide precision medicine.

## 10. Conclusions

Full activation of p53 function that leads to apoptosis and adaptive immune response culminating in immunogenic cell death is the Holy Grail of cancer therapy. Therefore, it is vital that novel discoveries and protocols, even in the realm of immuno-oncology, focus on strategies that will activate p53 and harness its power to eradicate cancer lesions, irrespective of the tissue of origin. Such an achievement will simplify the therapeutic approach and reduce the economic burden by treating large numbers of cancers that harbor wild-type or phenotypically functional mutant p53. To achieve this goal, it is critical to embrace that the functional response rather than the genotype of p53 is the more important indicator of therapeutic outcome. To complement this awareness, an effort has been made herein to better predict the functional status of p53 and therapeutic response by proposing FENOMP, which is distinct from the current predictive databases by providing an accurate predictive readout using the patient’s own tumor as the test model. Kastan in 2007 concluded that “tumors cannot stand” wild-type p53; almost two decades later it is time to re-validate the seminal role of p53 in cancer therapy and its value in precision medicine with the goal of reaffirming this biomolecule as the Achilles Heel of cancer in medical oncology.

## Data Availability

No new data were created or analyzed in this study.

## Abbreviations

The following abbreviations are used in this manuscript:

CLL	chronic lymphocytic leukemia
CTRD	C-terminal regulatory domain
DBD	DNA-binding domain
FASAY	functional analysis of separated alleles in yeast
FENOMP	functional evaluation of normal (wild-type) or mutant p53
FDA	The U.S. Food and Drug Administration
HGSOC	high-grade serous ovarian cancer
HRR	homologous recombination repair
NSCLC	non-small cell lung cancer
p53RE	p53 response element
PHANTM	phenotypic annotation of *TP53* mutations
PRD	proline-rich domain
qPCR	quantitative polymerase chain reaction (real-time PCR)
RING	really interesting new gene
Ser	serine
TetD	tetramerization domain
UV	ultraviolet
